# Generalization Bounds Derived IPM-Based Regularization for Domain Adaptation

**DOI:** 10.1155/2016/7046563

**Published:** 2015-12-27

**Authors:** Juan Meng, Guyu Hu, Dong Li, Yanyan Zhang, Zhisong Pan

**Affiliations:** College of Command Information System, PLA University of Science and Technology, Nanjing 210007, China

## Abstract

Domain adaptation has received much attention as a major form of transfer learning. One issue that should be considered in domain adaptation is the gap between source domain and target domain. In order to improve the generalization ability of domain adaption methods, we proposed a framework for domain adaptation combining source and target data, with a new regularizer which takes generalization bounds into account. This regularization term considers integral probability metric (IPM) as the distance between the source domain and the target domain and thus can bound up the testing error of an existing predictor from the formula. Since the computation of IPM only involves two distributions, this generalization term is independent with specific classifiers. With popular learning models, the empirical risk minimization is expressed as a general convex optimization problem and thus can be solved effectively by existing tools. Empirical studies on synthetic data for regression and real-world data for classification show the effectiveness of this method.

## 1. Introduction

The generalization ability is a main concern of statistical learning theory [1]. How to improve the predicting accuracy under the empirical risk minimization (ERM) principle has practical meaning since ERM-based learning process is widely used nowadays. As one important technique to improve generalization ability or avoid so-called overfitting, regularization plays a crucial role to maintain the trade-off of the empirical loss and the expected risk. Different regularizer may acquire different performance, and the choice depends on the specific purposes.

For traditional supervised learning, many labeled data are needed for training a precise model. It is well-known that annotating is both labour and time consuming with large amounts of unlabeled data. Another underlying assumption is that training data and testing data are separately provided while drawn from the same distribution; thus we can use the model trained on the former to predict labels of the latter, while the real situations we may always confront are that the available labeled data are from different sources and are different from what we need to predict. In other words, labeled data from target domain are not always accessible or sufficient. As a consequence, the provided labeled data cannot be trained directly to gain predictors on the target data.

As an efficient method to utilize small number of labeled data, or even unlabeled data from other sources, domain adaptation has obtained more attention in recent years [2–4]. Patterns from source domain and target domain are utilized to acquire better predictive ability on target data. Learning from multiple source domains [5] and combining source and target domains [6] are popular methods proposed in recent years. Along with some successful application related to domain adaptation, several works focused on the learning ability on this paradigm. Specifically, [7] studies the generalization bounds of domain adaptation, in which the integral probability metric (IPM) [8] is chosen to measure the distance between the source domain and the target domain. A natural idea is how to combine the theoretical results and the practical algorithm designing, thus creating more efficient learning algorithms.

In this paper, we proposed a framework for domain adaptation combining source and target data, taking the IPM as the regularization term. Since the IPM is defined as the upper bound of the gap between two distributions (source domain and target domain), the regularization term is independent with specific predictors. In other words, many popular learning models can be used under such a framework. For many cases, the empirical risk minimization problems could be solved efficiently as convex optimization problems in considerable times.

The remainder of this paper is organized as follows.  Section 2 reviews related works about theoretical analysis of domain adaptation problems and a regularized domain adaptation framework.  Section 3 introduced the problem set-up of and the derived IPM-based generalization bounds. We propose the framework in Section 4 and report the experimental results of regression and classification in Section 5.  Section 6 concludes this paper.

## 2. Related Works

There have been many works focused on the theoretical analysis of domain adaptation. Generally speaking, the generalization performance is measured by the size of training set, complexity of function class, and several constants. Specifically for domain adaptation, one also needs to measure the divergence of different distributions. For the complexity measurement of function class, VC-dimension is widely used in traditional learning model as well as in domain adaptation [4, 6, 9]. Besides VC-dimension, the covering number and Rademacher complexity are also used to measure the function class in generalization bounds of domain adaptation [5, 7]. In terms of the measurement of different distributions, *ℋ*-divergence is used in [4, 6]; the same concept is called *𝒜*-distance in [9] and derived from [10]. It was defined as the upper bound of two probability distributions, which is straightforward for classification. Both [5] and [7] introduce different quantities for more general tasks including regression, while the latter further take the labeling function into consideration.

One significant meaning of theoretical analysis is to provide guidance of designing new algorithms. Most of the above works give out the generalization bounds of domain adaptation to provide important properties of learning process for domain adaptation instead, such as convergence rate, effectiveness, and correctness.

In terms of regularized domain adaptation, a framework called domain adaptation machine (DAM) [11, 12] describes a data dependent regularizer, which is based on smoothness assumption and a relevance between source domain and target domain. The framework is similar to our method in some way, while the definition and optimization are different. DAM mainly stresses domain adaptation from multiple sources, while we care about domain adaptation combining source (including multiple sources) and target data, which has different empirical loss as well as regularizer. However, the one regularizer in DAM has close connection with ours and the details can be found in later discussion.

## 3. Domain Adaptation

### 3.1. Problem Description

In domain adaptation, the source domain and target domain are denoted by *𝒵* ^(*S*)^≔*𝒳* ^(*S*)^ × *𝒴* ^(*S*)^ and *𝒵* ^(*T*)^≔*𝒳* ^(*T*)^ × *𝒴* ^(*T*)^. Distributions over input space *𝒳* ^(*S*)^ and *𝒳* ^(*T*)^ are donated by *𝒟* ^(*S*)^ and *𝒟* ^(*T*)^, respectively. Traditional supervised learning aims to learn a function *f* : *𝒳* ^(*T*)^ → *𝒴* ^(*T*)^ for labeling unseen samples in *𝒟* ^(*T*)^. In the domain adaptation set-up, *𝒟* ^(*T*)^ is hard to estimate directly with insufficient *𝒳* ^(*T*)^. With considerable amounts of *𝒳* ^(*S*)^ and *𝒴* ^(*S*)^, the minimization empirical risk over loss function *ℓ*(∘) with parameter vector *θ* can be expressed as follows:

(1) $$\begin{array}{c} \underset{\theta }{\min}\;E_{\theta }^{\left(S\right)}f=\frac{1}{N}\overset{N}{\underset{n=1}{\sum }}l\left(\;\theta ;x_{n}^{\left(S\right)},y_{n}^{\left(S\right)}\;\right), \end{array}$$

where *E* ^(*S*)^ is the expectation taken with respect to the distributions *𝒵* ^(*S*)^. In order to utilize more information of target domain, available target samples should be used. Given *τ* ∈ [0,1), domain adaptation combining source and target data is defined to minimize the empirical risks [4]:

(2) $$\begin{array}{c} E_{\tau }f=\tau E^{\left(T\right)}f+\left(\;1-\tau \;\right)E^{\left(S\right)}f, \end{array}$$

where *τ* controls the trade-off between learning from source data and target data.

### 3.2. Integral Probability Metric

In domain adaptation, it is important to find a quantity measuring the difference of the distributions between the source and the target domains. In this paper, we use the integral probability metric (IPM) to measure the difference between two distributions. This quantity is defined as the distance between the source domain *𝒵* ^(*S*)^ and the target domain *𝒵* ^(*T*)^, under function class *ℱ* ⊂ *ℝ* ^*𝒵*^:

(3) $$\begin{array}{c} D_{F}\left(\;S,T\;\right)≔\underset{f\in F}{\sup}\left|\;E^{\left(S\right)}f-E^{\left(T\right)}f\;\right|. \end{array}$$

The quantity *D*_*ℱ*_(*S*, *T*) is aimed at measuring the difference between the two probability distributions. If the source domain *𝒵* ^(*S*)^ and the target domain *𝒵* ^(*T*)^ have the same probability distribution, the quantity *D*_*ℱ*_(*S*, *T*) is equal to zero.

Assuming there are *N*_*S*_ samples drawn from source domain and *N*_*T*_ samples from target domain, the expectations *E* ^(*S*)^ *f* and *E* ^(*T*)^ *f* can be roughly estimated by these samples; thus the *D*_*ℱ*_(*S*, *T*) can be approximated by the expectations over given data. However, the target samples are not enough to learn a predictor; that is, *N*_*T*_ ≪ *N*_*S*_; then domain adaptation minimize the convex combination of the source and the target empirical risk, for *τ* ∈ [0,1),

(4) $$\begin{array}{c} E_{\tau }f≔\tau E_{N_{T}}^{\left(T\right)}f+\left(\;1-\tau \;\right)E_{N_{S}}^{\left(S\right)}f. \end{array}$$

When *τ* = 0, it provides a learning process of the basic domain adaptation with one single source.

### 3.3. Generalization Bounds

The generalization bounds of a learning process need to consider three essential aspects: complexity measure of function class, Hoeffding-type deviation inequality, and symmetrization inequality.

Different from the classical VC-dimension form, Zhang et al. [7] chose the uniform entropy number to measure the complexity which is derived from the concept of the covering number [13]. The covering number is denoted by *𝒩*(*ℱ*, *ϵ*, *d*), where *ℱ* is the function class, *d* is a metric on *ℱ*, and the covering number of *ℱ* at radius *ϵ* with respect to *d* is the minimum size of a cover of radius *ϵ*. The covering number is not suitable for domain adaptation. As a variant of the covering number, by setting the metric *ℓ*_1_^*τ*^(*Z*), the uniform entropy number is defined as follows:

(5) $$\begin{array}{c} \ln N_{1}^{\tau }\left(\;F,\epsilon ,2\left(\;N_{S}+N_{T}\;\right)\;\right)≔\underset{Z}{\sup}\ln N\left(\;F,\epsilon ,l_{1}^{\tau }\left(\;Z\;\right)\;\right). \end{array}$$

The uniform entropy number is distribution-free and can be chosen as the complexity measure of function class to derive the generalization bounds for domain adaptation.

Hoeffding-type deviation inequality for domain adaptation is an extension of the classical Hoeffding-type deviation inequality which allows the random variables to take values from different domains. It is assumed that *ℱ* is a function class consisting of bounded functions with the range [*a*, *b*]. A function *F*_*τ*_ is defined as follows:

(6) FτX1NT,Y1NS≔τNS∑n=1NTfxn+1−τNT∑n=1NSfyn.

For any *τ* ∈ [0,1) and any *ξ* > 0,

(7) Pr⁡FτZ1NS,Z1NT−E∗Fτ>ξ≤2exp⁡−2ξ2b−a2NSNT1−τ2NT+τ2NS,

where the expectation *E* ^(*∗*)^ is taken on both the source domain *Z* ^(*S*)^ and the target domain *Z* ^(*T*)^.

Symmetrization inequality for domain adaptation has a discrepancy term (1 − *τ*)*D*_*ℱ*_(*S*, *T*) compared to the classical symmetrization result under the assumption of the same distribution. For any *ξ* > (1 − *τ*)*D*_*ℱ*_(*S*, *T*), the probability of the event

(8) $$\begin{array}{c} \underset{f\in F}{\sup}\left|\;E^{\left(T\right)}f-E_{\tau }f\;\right|>\xi \end{array}$$

can be bounded by using the probability of the event

(9) $$\begin{array}{c} \underset{f\in F}{\sup}\left|\;E_{\tau }^{\prime }f-E_{\tau }f\;\right|>\frac{\xi^{\prime }}{2}, \end{array}$$

where *ξ*′ = *ξ* − (1 − *τ*)*D*_*ℱ*_(*S*, *T*).

Based on the uniform entropy number, using a specific Hoeffding-type deviation inequality and symmetrization inequality, the generalization bounds of domain adaptation combining source and target data are derived as follows.

Assume that *ℱ* is a function class consisting of the bounded functions with the range [*a*, *b*]. For any *τ* ∈ [0,1) and given an arbitrary *ξ* > (1 − *τ*)*D*_*ℱ*_(*S*, *T*), we have, for any *N*_*S*_ *N*_*T*_ ≥ 8(*b* − *a*)^2^/*ξ* ^′2^, with probability of at least 1 − *ϵ*,

(10) supf∈F⁡Eτf−ETf≤1−τDFS,T+ln⁡NτF,ξ′/8,2NS+NT−ln⁡ϵ/8NSNT/32b−a21−τ2NT+τ2NS1/2.

The derived bound contains a term of discrepancy quantity (1 − *τ*)*D*_*ℱ*_(*S*, *T*).

## 4. IPM-Based Regularization Framework

From formula (10), we can see that the generalization bounds of domain adaptation consisted of two parts: integral probability metric (IPM) and the extension of the covering number (referred to as the uniform entropy number). Since the IPM is relatively easy to compute with source data and target data available, it is straightforward to take this term into regularization to reduce generalization error. Besides, it is also intuitive to make full use of target information to construct predictors. For single source, given data **X** ∈ *ℝ* ^*N*×*d*^ and corresponding label (or target value for regression) **y** ∈ *ℝ* ^*N*^, take *θ* ∈ *ℝ* ^*d*^ as the parameters of model and *ℓ*(*θ*; **x**, *y*) as the loss of a single sample. The general objective function for supervised learning can be written in the following risk minimization problem:

(11) $$\begin{array}{c} \underset{\theta }{\min}\frac{1}{N}\overset{N}{\underset{n=1}{\sum }}l\left(\;\theta ;x_{n},y_{n}\;\right)+\lambda R\left(\;\theta \;\right), \end{array}$$

where *R*(*θ*) is the regularizer and *λ* is the balancing parameter.

Based on the definition of IPM (3), empirical risk (4), and learning principle (11), we formally propose the framework of domain adaptation combining the source and the target data by replacing the regularizer. Consider

(12) $$\begin{array}{c} \underset{\theta }{\min}\;\tau E^{\left(T\right)}f+\left(\;1-\tau \;\right)E^{\left(S\right)}f+\lambda \left(\;1-\tau \;\right)D_{F}\left(\;S,T\;\right), \end{array}$$

where *Ef* = (1/*N*)∑_*n*=1_^*N*^ *ℓ*(*θ*; **x**_*n*_, *y*_*n*_).

In [14], the IPM can be empirically estimated by various popular distance metrics by appropriately choosing *ℱ*. Specifically in the reproducing kernel Hilbert space (RKHS), IPM is called kernel distance or maximum mean discrepancy (MMD) [15]. The empirical estimator of MMD is straightforward:

(13) MMDF,S,T=1Ns∑n=1NsϕxnS−1NT∑n=1NTϕxnTH,

where *ϕ* : *𝒳* → *ℋ* is called a feature space mapping function and two feature maps are defined as the kernel, *k*(**x** ^(*S*)^, **x** ^(*T*)^) = 〈*ϕ*(**x** ^(*S*)^), *ϕ*(**x** ^(*T*)^)〉.

DAM frameworks [12] construct a domain-dependent regularizer for domain adaptation from multiple sources, which is defined as

(14) $$\begin{array}{c} \Omega \left(\;f^{T}\;\right)=\frac{1}{2}\overset{P}{\underset{s=1}{\sum }}\gamma_{s}{\left\|\;f_{u}^{T}-f_{u}^{S}\;\right\|}^{2}, \end{array}$$

where *P* is the number of source domains, **f**_*u*_^*T*^ and **f**_*u*_^*S*^ are the decision values from the target classifier, and the *s*th classifier on the unlabeled instances in the target domain. Here the coefficient *γ*_*s*_ is set as exp(−*β* × MMD[*ℱ*, *S*, *T*]^2^).

From the definition we can see that the regularizer we use in (12) is much simpler than that in DAM. Moreover, the objective function in DAM consists of three parts, other two include the regularizer which controls the complexity of target classifier and the loss of target classifier, while the objective function we use in (12) considers a combination of the loss over source domain and target domain [4].

The proposed framework is also suitable for domain adaptation combining multiple sources, where *E* ^(*S*)^ *f* and regularization term *D*_*ℱ*_(*S*, *T*) in (12) are defined as a linear combination of several terms. Consider

(15) ESf=∑i=1PwiEiSf=∑i=1PwiNi∑n=1Nilθ;xni,yni,

(16) DFS,T=∑i=1PwiDFSi,T.

The generalization bound of domain adaptation from multiple sources has similar form with (10), where the first term on the right side is a linear combination of several IPMs instead of one; see (16).

## 5. Experiments

We first carry out experiments on both simple regression and classification problems to verify the effectiveness of (12). For the purpose of easy-to-optimize, we use least square *ℓ*(*θ*; **x**, *y*) = (**x** ^*T*^ *θ* − *y*)^2^ as the loss function. It is straightforward in regression since the target value is continuous, while for binary classification there are a few articles that discussed this loss. Reference [16] employed it in text classification and [17] pointed out the rationality of least square loss compared with SVM. Since the loss is quadratic while the IPM is expressed as an absolute value under this setting, it is necessary to convert the regularizer into the squared form of the original value to balance these two terms, and it can be approximated by the gap of losses on target domain and source domain, that is, (*E* ^(*S*)^ *f* − *E* ^(*T*)^ *f*)^2^. All these tricks make the whole objective function consisting of both loss function and regularizer convex much easier to optimize. We use the limited-memory BFGS provided by package yagtom (https://code.google.com/p/yagtom/) in experiments.

In the last part of experiment, we would apply least squares support vector machine (LS-SVM) [18] as the classifier; the loss function is expressed as *ℓ*(*θ*; **x**, *y*) = (*θ* ^*T*^ *ϕ*(**x**) − *y*)^2^, where *ϕ*(·) is the kernel function. Regularization for LS-SVM is commonly used, *R*(*θ*) = *C*‖*θ*‖^2^, where parameter *C* controls the balance.

### 5.1. Regression

We perform numeric experiments on synthetic data for regression test and only consider single source. For target domain, we assume **X** ^(*T*)^ ∈ *ℝ* ^*N*×100^ from a Gaussian distribution *N*(0,1) and the noise vector **R** ∈ *ℝ* ^*N*^ with *N*(0,0.5); let model parameters vector *θ* ∈ *ℝ* ^100^ of *N*(1,5); then the target values are generated by

(17) $$\begin{array}{c} y_{n}^{\left(T\right)}=\langle \;x_{n}^{\left(T\right)},\theta \;\rangle +R. \end{array}$$

The derived (**x**_*n*_^(*T*)^, *y*_*n*_^(*T*)^)_*n*=1_^*N*_*T*1_^ will be used in training and cotraining with data from source domain, and (**x**_*n*_^(*T*)^, *y*_*n*_^(*T*)^)_*n*=1_^*N*_*T*_^  (*N*_*T*_ = 2000) will be used as the test data. Similarly, the sample set (**x**_*n*_^(*S*)^, *y*_*n*_^(*S*)^)_*n*=1_^*N*_*S*_^  (*N*_*S*_ = 2000) will be used as source domain and the generating rule is

(18) $$\begin{array}{c} y_{n}^{\left(S\right)}=\langle \;x_{n}^{\left(S\right)},\theta \;\rangle +R, \end{array}$$

where **x**_*n*_^(*S*)^ ~ *N*(0,0.2), *θ* ~ *N*(1,5), and **R** ~ *N*(0,0.5).

With the fitting accuracy root mean squared error (RMSE) as the criterion, we conducted the following four settings in the experiments:

(i) setting 1, *N*_*T*1_ → *N*_*T*_: training on the small parts of target domain (*N*_*T*1_) and testing on target domain (*N*_*T*_);
(ii) setting 2, *N*_*S*_ → *N*_*T*_: training on the source domain (*N*_*S*_) and testing on target domain (*N*_*T*_);
(iii) setting 3, *N*_*S*_ + *N*_*T*1_ → *N*_*T*_: training on the source domain (*N*_*S*_) combining small parts of target domain (*N*_*T*1_) and testing on target domain (*N*_*T*_);
(iv) setting 4, *N*_*S*_ + *N*_*T*1_ + IPM → *N*_*T*_: training on the source domain(*N*_*S*_) combining small parts of target domain (*N*_*T*1_) with regularizer and testing on target domain (*N*_*T*_).

We search the parameter *λ* in range of [2^−10^, 2^−9^,…, 2^10^] in setting 4 and *τ* = (*N*_*T*1_)/(*N*_*T*1_ + *N*_*S*_) in setting 3 and setting 4 according to the similar numeric experiments to evaluate the asymptotic convergence in [7]. 10 rounds for each problem have been conducted and the average of RMSE is recorded as the result. All the results are shown in Table 1.

We can see, in all cases, that RMSE in setting 4 is the smallest. It makes sense to say that the domain adaptation with the IPM regularizer can obtain better performance than without it.

### 5.2. Classification

When adopting square loss function in binary classification, we require the sample *x*_*n*_'s label *y*_*n*_ ∈ {−1,1}. Assume the output label of **x**_*n*_ is ${\hat{y}}_{n}=x_{n}^{T}\theta$; in case that ${\hat{y}}_{n}*y_{n}>0$ the predicting is right.

The binary classification tests are carried on text datasets email spam (available at http://www.ecmlpkdd2006.org/challenge.html) and parts of 20 newsgroups datasets (http://vc.sce.ntu.edu.sg/transfer_learning_domain_adaptation/). The email spam dataset contains a set of 4000 public labeled emails which is used here as target domain data and other three sets, each of which has 2500 emails annotated by different users and would be used as source domain data. In these four datasets, samples are labeled as nonspam (*y* = 1) or spam emails (*y* = −1). The 20 newsgroups datasets recollected by Duan et al. [12] contains three groups and each has a target set with three sources. Details of the datasets used in classification are shown in Table 2.

So we have 12 groups of source-target pairs in total to conduct the experiments; in each pair we randomly choose *N*_*T*1_ = 20 samples from the target domain to participate in domain adaptation and the classification accuracy on the rest target set is chosen as the evaluation criterion. The parameters *λ* and *τ* are picked in the same way as in the regression experiment, and result in each pair is averaged over 10 times running. The comparison of classification accuracy is listed in Table 3.

As we can see, the domain adaptation with the IPM regularizer can obtain better performance than without it and is even better than just training on small target domain samples in most cases.

### 5.3. Classification with LS-SVM

In order to improve the classification ability in real datasets, we adopt LS-SVM with kernel as the predictor. The square of MMD is easily obtained by (19), by expanding the original definition. Here in the experiments we use linear kernel for convenience of getting MMD (13); that is, *k*(**x** ^(*S*)^, **x** ^(*T*)^) = (**x** ^(*S*)^)^*T*^ **x** ^(*T*)^. What is more, the regularization term is independent with the model parameters. Consider

(19) MMDF,S,T2=1NS2∑i,jNSkxiS,xjS+1NT2∑i,jNTkxiT,xjT−2NSNT∑i,j=1NS,NTkxiS,xjT.

**Figure 1:**
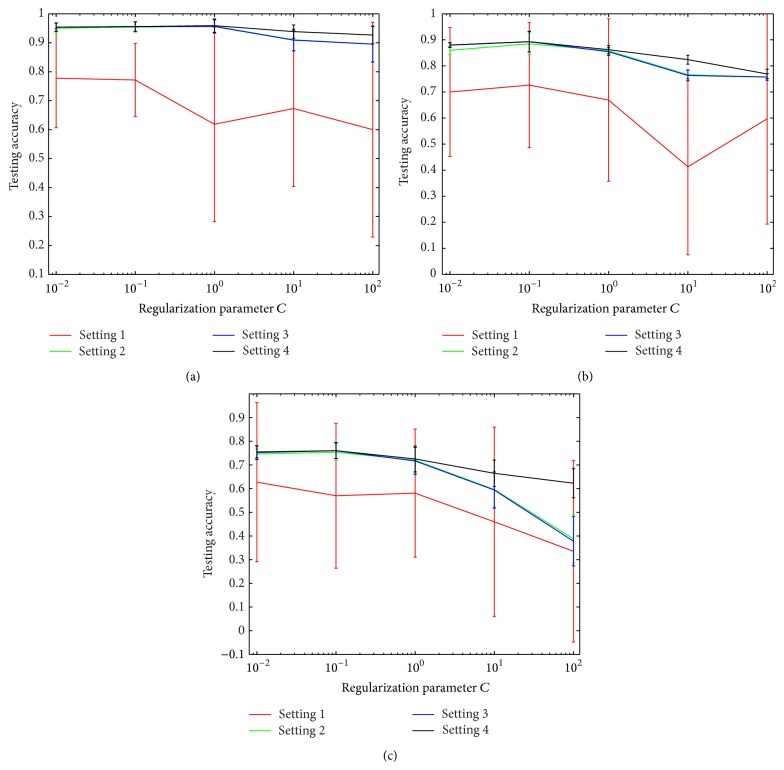
Comparison of testing accuracy and standard deviations on 20 newsgroups datasets, with each problem setting parameter *C* = $\left[\begin{array}{ccccc} 0.01 & 0.1 & 1 & 10 & 100 \end{array}\right]$. The number of labeled data from target domain is 10. From (a) to (c): comp versus rec, rec versus sci, and sci versus comp.

In this part, we adopt a paradigm of domain adaptation combining multiple sources. As a consequence, in settings 2, 3, and 4, the risk on source domain is computed by (15) and in setting 4 the regularization term IPM is computed by (16) and (19). In each problem, there are three sources. First of all, we search the regularization parameter *C* in single LS-SVM predictor, that is, *R*(*θ*) = *C*‖*θ*‖^2^ of (11), in range $\left[\begin{array}{ccccc} 0.01 & 0.1 & 1 & 10 & 100 \end{array}\right]$, on the 20 newsgroups datasets. We can see from Figure 1 that the proposed method tends to achieve best testing accuracy and low standard deviations. In all datasets with any value of *C*, setting 1 has the lowest testing accuracy and relatively high standard, due to the insufficient training with small amounts of labeled data. As in most cases, *C* = 0.1 has the best performance; we set this value in the following experiments.

All results on the same datasets listed in Table 2 are shown in Table 4. We can see that in most cases, the proposed algorithm outperformed other methods from a statistical perspective. Setting 1 had the worst accuracy, which means training on small amounts of target data is not sufficient. The fact that accuracy in setting 1 increases as the available labeled data becomes more, which fits the experience of ERM learning. It seems that the performance of setting 2 is even slightly better than setting 3 in most cases; thus simply combining risks over source and target domain to learn may not work in practice. On the other hand, the IPM regularization term does provide a bridge between this gap.

## 6. Conclusion

In this paper, we proposed a general framework for regularized domain adaptation combining source(s) and target data. The regularization mainly considers the gap between source domain and target domain and uses the integral probability metric as the distance measurement of different domains. Square approximation and inner product in RKHS tricks are used for empirical estimation of the IPM. The IPM regularization term is supposed to reduce the generalization error according to a theoretical work [7]. The regularization method can work for domain adaptation combining single source as well as multiple sources, and a sort of popular predictor can be utilized. Experiments on regression and classification indicate that this method can work better than original domain adaptation without the regularization term.

We are also interested in the relationship between semisupervised learning and domain adaptation with few labeled target domain samples, since they share similar problem settings. And for cases when labeled target data is unavailable, the obtained pseudolabel may help. Theoretical analysis and empirical results are going to be investigated.

## Figures and Tables

**Table 1:** The comparison of RMSE on four settings with different labeled target domain samples.

*N*_*T*1_	Setting 1	Setting 2	Setting 3	Setting 4
20	42.7473	0.7642	0.7546	** 0.7175**
50	34.1598	0.7639	0.7312	** 0.6894 **
100	7.9272	0.7594	0.6690	** 0.6495 **
200	0.7249	0.7640	0.6071	** 0.5812 **

**Table 2:** Description of the email spam dataset and 20 newsgroups datasets [12].

	Source domains (*N*_*S*_)	Target domains (*N*_*S*_)
Email spam	User 1 (2500)	Public set (4000)
	User 2 (2500)	
	User 3 (2500)	

rec versus sci	rec.autos and sci.crypt (1976)	rec.sport.hockey and sci.space (1982)
	rec.motorcycles and sci.electronics (1977)	
	rec.sport.baseball and sci.med (1978)	

comp versus rec	comp.graphics and rec.autos (1957)	comp.sys.mac.hardware and rec.sport.hockey (1955)
	comp.os.ms-windows.misc and rec.motorcycles (1956)	
	comp.sys.ibm.pc.hardware and rec.sport.baseball (1970)	

sci versus comp	sci.crypt and comp.graphics (1959)	sci.space and comp.sys.mac.hardware (1943)
	sci.electronics and comp.os.ms-windows.misc (1947)	
	sci.med andcomp.sys.ibm.pc.hardware (1966)	

**Table 3:** The comparison of classification accuracy, *N*_*T*1_ = 20.

Dataset	Setting 1	Setting 2	Setting 3	Setting 4
Email spam	0.6686	0.7625	0.6962	** 0.8037**
	0.5681	0.6514	0.6962	**0.8138 **
	0.7461	0.7972	0.6962	**0.8078 **

20 newsgroups: comp versus rec	0.7051	0.8525	0.5885	**0.8848**
	0.8132	0.8806	0.5885	**0.9017 **
	0.9452	0.9466	0.5885	**0.9551 **

20 newsgroups: rec versus sci	0.6117	0.7849	**0.8329**	0.7942
	0.7205	0.8432	0.8329	**0.8530 **
	0.8623	0.9036	0.8329	**0.9293 **

20 newsgroups: sci versus comp	0.7142	0.7875	0.6062	**0.8078 **
	0.5295	0.5868	**0.6062**	0.5818
	0.8255	0.8550	0.6062	**0.8853 **

**Table 4:** The comparison of classification accuracy (LS-SVM), with multiple sources.

Dataset	Setting 1	Setting 2	Setting 3	Setting 4
*N*_*T*1_ = 5				
Email spam	0.6457 ± 0.0828	0.9258 ± 0.0084	0.9251 ± 0.0081	0.9371 ± 0.0129
20 newsgroups: comp versus rec	0.6020 ± 0.3116	0.9498 ± 0.0234	0.9479 ± 0.0253	0.9509 ± 0.0226
20 newsgroups: rec versus sci	0.5922 ± 0.4323	0.8315 ± 0.0184	0.8287 ± 0.0183	0.8427 ± 0.0201
20 newsgroups: sci versus comp	0.4887 ± 0.3959	0.6988 ± 0.0469	0.6947 ± 0.0476	0.7106 ± 0.0474

*N*_*T*1_ = 10				
Email spam	0.7211 ± 0.0812	0.9274 ± 0.0075	0.9269 ± 0.0073	0.9337 ± 0.0057
20 newsgroups: comp versus rec	0.6119 ± 0.3517	0.9596 ± 0.0212	0.9581 ± 0.0229	0.9594 ± 0.0217
20 newsgroups: rec versus sci	0.6173 ± 0.3125	0.8485 ± 0.0225	0.8481 ± 0.0216	0.8507 ± 0.0228
20 newsgroups: sci versus comp	0.5135 ± 0.3056	0.7485 ± 0.0557	0.7455 ± 0.0579	0.7508 ± 0.0549

*N*_*T*1_ = 15				
Email spam	0.7465 ± 0.0644	0.9201 ± 0.0171	0.9195 ± 0.0167	0.9231 ± 0.0179
20 newsgroups: comp versus rec	0.7206 ± 0.2425	0.9487 ± 0.0225	0.9467 ± 0.0239	0.9478 ± 0.0232
20 newsgroups: rec versus sci	0.5872 ± 0.3634	0.8443 ± 0.0116	0.8427 ± 0.0103	0.8490 ± 0.0128
20 newsgroups: sci versus comp	0.5286 ± 0.2284	0.7294 ± 0.0574	0.7270 ± 0.0591	0.7354 ± 0.0518

*N*_*T*1_ = 20				
Email spam	0.7786 ± 0.0578	0.9286 ± 0.0036	0.9279 ± 0.0033	0.9309 ± 0.0045
20 newsgroups: comp versus rec	0.7760 ± 0.2496	0.9543 ± 0.0173	0.9526 ± 0.0183	0.9545 ± 0.0172
20 newsgroups: rec versus sci	0.7536 ± 0.1642	0.8618 ± 0.0121	0.8626 ± 0.0120	0.8626 ± 0.0120
20 newsgroups: sci versus comp	0.6867 ± 0.2298	0.7006 ± 0.0624	0.6963 ± 0.0660	0.7016 ± 0.0631
